# Pathogenic diversity of *Klebsiella pneumoniae* strains with different serotypes and sequence types from human liver abscess

**DOI:** 10.3389/fmicb.2026.1730966

**Published:** 2026-03-04

**Authors:** Xue Ren, Xuanfeng Liu, Yujie Chen, An Su, Bing Du, Hanqing Zhao, Yanling Feng, Guanhua Xue, Jinghua Cui, Yuehua Ke, Lin Gan, Junxia Feng, Zheng Fan, Tongtong Fu, Ziying Xu, Zihui Yu, Yang Yang, Tingting Zhang, Lei Huang, Chao Yan, Jing Yuan

**Affiliations:** 1 Capital Center for Children’s Health, Capital Medical University, Capital Institute of Pediatrics, Beijing, China; 2 Capital Institute of Pediatrics, Chinese Academy of Medical Sciences & Peking Union Medical College, Beijing, China; 3 Capital Institute of Pediatrics-Peking University Teaching Hospital, Beijing, China; 4 Capital Center for Children’s Health, Capital Medical University, Beijing, China; 5 Center for Infectious Diseases, The Fifth Medical Center of Chinese PLA General Hospital, Beijing, China

**Keywords:** *Klebsiella pneumonia*, liver abscess, pathogenicity, sequence type, serotype

## Abstract

**Background:**

*Klebsiella pneumoniae* (*K. pneumoniae*) strains exhibit diverse virulence phenotypes influenced by serotype (K-type) and sequence type (ST). However, the interplay between bacterial molecular/biological characteristics and specific pathogenicity remains poorly understood.

**Methods:**

We characterized 11 clinical *K. pneumoniae* isolates from liver abscess patients with varying serotypes (K1, K2, K5, K20, K57, K80), STs (including novel variants), and virulent factors. Phenotypic assays included the determination of growth curves, assessment of biofilm formation, and observation via electron microscopy. The Vitek 2 automated system was used to evaluate the minimum inhibitory concentration (MIC) of 11 clinical *Klebsiella pneumoniae* strains against common antibiotics. Mouse infection models were used to assess survival rates, analyze organ pathology, and detect hematological changes; these experimental data were complemented by clinical patient data.

**Results:**

Eleven clinically isolated *Klebsiella pneumoniae* strains displayed serotype/genotype-associated virulence and short bacilli morphology under TEM, with seven being hypermucoviscosity-positive. Growth and biofilm phenotypes varied: K2-ST65 (S2-029) had superior proliferation, K2-ST86 (S2-048) the weakest growth; K1-ST23 (S1-001) formed the strongest biofilm, while K57-ST218 (S57-077) the weakest. Phylogenetic analysis based on core SNPs derived from whole-genome sequencing of 11 *K. pneumoniae* strains revealed that S5-105 and S5-036 formed a distinct independent clade, significantly separated from the remaining strains. Notably, S5-105 and S5-036 exhibited significantly higher levels of differentiation compared to the other strains. Antibiotic susceptibility testing showed 9/11 of strains were fully sensitive, with overall low resistance. All strains were 100% susceptible to carbapenems, cephalosporins, aminoglycosides, and aztreonam. Only S5-105 was ciprofloxacin-resistant/levofloxacin-intermediate among quinolones; two strains had sulbactam-related non-susceptibility (S5-036: intermediate; S57-066: piperacillin-intermediate + sulbactam-resistant). Notably, two novel ST-type strains exhibited unique pathogenicity: K1-novel ST (S1-009) caused rapid systemic infection, 50% 72 h survival, severe liver abscess, and neutrophilic inflammation; K80-novel ST selectively induced pulmonary abscesses without hepatic involvement, an atypical tropism. K2 strain virulence correlated with ST: hypervirulent K2-ST65 led to 50% survival, while K2-ST86 resulted in 100% survival. Other serotypes showed distinct pathogenicity: K20 caused liver damage, K57 was nearly non-pathogenic, and slow-growing K5 induced mild tissue injury.

**Conclusion:**

Specific serotype-STs combination potentiates lethality under certain circumstances (e.g., K2-ST65), but its impact is complicated. Novel STs correlate with acute lethality (K1) or atypical tropism (K80 lung—specific virulence). These findings highlight strain—specific virulence mechanisms, which are critical for the risk stratification of *K. pneumoniae* infection.

## 1 Introduction

In recent years, *Klebsiella pneumoniae* (*K. pneumoniae*/Kpn) infections have shown a global spread. Multiple studies conducted in China have indicated a significant increase in its prevalence: Among elderly patients from 2008 to 2014, *K. pneumoniae* accounted for 45.7% of infection, with cases doubling annually (Liu and Guo, 2019). In 2016, the proportion of *K. pneumoniae* infections in Wenzhou reached as high as 68.8%, and liver abscess (LA) was identified as a major risk factor for infection [odds ratio (OR) = 10.154] (Hao et al., 2020). Originally concentrated in Asia, *K. pneumoniae* infections have now expanded to Europe and the Americas. For instance, a hospital in New York detected 17 cases of community—acquired *K. pneumoniae* infections over a 3 years period; these strains tested positive for virulence genes and were associated with high mortality rates (Parrott et al., 2021). This evidence demonstrates that *K. pneumoniae* has evolved from a regional pathogen into a global health threat, demanding increased attention.

The specific characteristics of *K. pneumoniae* are closely linked to the incidence and mortality of LA, with K1/K2 capsular serotypes being key virulence determinants. In Asian regions, K1/K2 serotypes exhibit a dominant distribution pattern in *K. pneumoniae*—related LA infections. A study in Inner Mongolia, China, found that among 78 patients with *K. pneumoniae*—derived LA, K1 and K2 serotypes accounted for 56.4 and 26.9% of isolates, respectively (Li et al., 2023). Research in Taiwan further revealed that K1 capsular type predominated among primary LA isolates; all these K1 strains universally carried virulence-associated plasmid genes, such as *rmpA* and *aerobactin*, exhibiting a detection rate of 100% (Yu et al., 2008).

Molecular epidemiological studies have revealed correlations between specific genotypic combinations and clinical outcomes. The K1-ST23 clone is significantly associated with LA cases worldwide. In Eastern China, studies showed that 57.8% of LA—related *K. pneumoniae* strains belonged to ST23—type, and 96.2% of these strains were simultaneously positive for *magA* and exhibited K1 serotype characteristics (Qu et al., 2015). In contrast, K2-ST65 strains demonstrate stronger phenotypic heterogeneity, and their virulence potentially linked to ethanol production (Cubero et al., 2016). Notably, in patients with alcoholic liver disease, infection with high-alcohol-producing *Kpn* strains creates a vicious cycle, which significantly exacerbates liver inflammatory damage and leads to a marked increase in mortality rates, suggesting that ethanol may disturb this disease process.

In addition to the K1/K2 capsular types, the role of non-classical capsular types, such as K20, K57, and K80, in the pathogenesis and prognosis of liver abscess has gradually attracted attention. Studies on different capsular types have found that K57 and K80 strains may possess unique pathogenic mechanisms. Although specific data on their lethality remain limited, genomic characteristics suggest that strains carrying virulence genes such as *rmpA* and *aerobactin* may exhibit strong virulence due to the conservation of these genes (Jiang et al., 2024). In some clinical cases, patients infected with novel ST-type *K. pneumoniae* strains may present with more complex clinical manifestations and poorer prognoses; this could be attributed to the combination of virulence genes and differences in host immune responses. Notably, with the increasing occurrence of virulence gene recombination events, certain atypical serotype strains may be developing new pathogenic features—posing novel challenges for the clinical diagnosis and treatment strategies of LA.

In the present study, we compared 11 *K. pneumoniae* strains with distinct molecular and biological characteristics from clinical samples of LA patients. These strains belonged to diverse sequence types (including ST23, ST65, etc.) and serotypes (K1/K2 and atypical serotypes such as K20, K57). A mouse model of LA infection was established to systematically evaluate the impact of these bacterial pathogenicity. The findings of this study not only contribute to elucidating the mechanistic roles of different genomic characteristics in the development of liver abscess but also provide crucial experimental evidence for guiding clinical treatment strategies.

## 2 Materials and methods

### 2.1 Specimen collection and isolation of *Klebsiella pneumoniae*

Eleven *Klebsiella pneumoniae* strains with different serotypes and/or sequence types were isolated from clinical patients with liver abscess, aimed to investigate their pathogenic potential characteristics and aggressive organ tropism. The *K. pneumoniae* reference strain NTUH-K2044 (GCA_000009885.1), and the 11 isolates were cultured in YPD (yeast extract peptone dextrose) medium. This study was approved by the medical ethics committee of the Capital Institute of Pediatrics.

### 2.2 Genome sequencing and ST analysis

For genomic comparison and analysis, 11 *K. pneumoniae* isolates were subjected to whole-genome sequencing. The sequencing was carried out on the Illumina HiSeq PE150 platform by the Institute of Microbiology, Chinese Academy of Sciences. The genome sequences were assembled using SOAP *de novo* (version 2.04), and gene annotation was performed with Prokka (version 1.14.6).

To determine the sequence types (STs), multilocus sequence typing (MLST) was applied. The sequences of the housekeeping genes *gapA*, *infB*, *mdh*, *pgi*, *phoE*, *rpoB*, and *tonB* were submitted to the Institut Pasteur *K. pneumoniae* MLST database,^1^ and the corresponding STs were identified (Diancourt et al., 2005).

### 2.3 Virulence genes analysis

Virulence factor identification was performed on 11 *K. pneumoniae* isolates using VFanalyzer (Virulence Factors of Pathogenic Bacteria)^2^ with NTUH-K2044 designated as the reference strain (Liu et al., 2019). This database catalogs key virulence determinants for *Klebsiella*, including genes associated with adherence, biofilm formation, efflux pumps, immune evasion, iron acquisition, nutritional factors, regulation, secretion systems, serum resistance, and toxins (Paczosa and Mecsas, 2016).

### 2.4 Phylogenetic population analysis

SNP (single nucleotide polymorphism) mainly refers to the polymorphism of DNA sequences caused by variations in a single nucleotide at the genomic level, including transitions and transversions of a single base, etc. SNPs were identified for 11 *K. pneumoniae* isolates using the MUMmer (Version 3.23) alignment software, with NTUH-K2044 as the reference genome (Croucher et al., 2015; Gan et al., 2022), and the functions of SNPs were annotated according to the positional relationship and interaction between SNPs and genes. The phylogenetic tree was constructed by the TreeBeST (Version 1.9.2) (Neighbor-Joining, NJ) or PhyML (Maximum likelihood, ML) (Version v3.0).

### 2.5 Antibiotic susceptibility testing

Antimicrobial susceptibility testing was performed using the Vitek 2 automated analysis system with AST-GN Gram-negative bacterial susceptibility cards, strictly following the manufacturer’s operating procedures. The detection range of minimum inhibitory concentration (MIC) for clinically commonly used antibacterial drugs was set. Bacterial suspensions were prepared using 0.45% normal saline and adjusted to a 0.5 McFarland standard. After loading the bacterial suspensions onto the susceptibility cards, the cards were placed in the Vitek 2 instrument for incubation of 18–24 h, during which the instrument automatically read the MIC values and susceptibility results. Bacterial suspensions were prepared from the same subculture plate, and the concentration and purity of the bacterial suspensions were verified by plating on blood plates free of antibacterial agents. Quality control strain (*Klebsiella pneumoniae* NTUH-K2044) was included throughout the test to ensure the reliability of the experimental results.

### 2.6 String test

The freshly cultured colony grown on a YPD plate is gently lifted using an inoculation loop by vertically streaking upward three times. If the stretched viscous filament exceeds 5 mm in length upon each pull, the test result is recorded as positive.

### 2.7 Growth curve and biofilm formation determination

The *K. pneumoniae* strain was cultured with YPD medium and its optical density at 600 nm (OD_600_) was measured at 1 h intervals. For biofilm formation analysis, the strain was diluted (1:50) with fresh medium. The bacterial suspension was then added to a 96-well microplate and incubated for 24 h under 37°C. After incubation, non-adherent bacteria were removed, and the formed biofilms were stained with crystal violet solution. The absorbance of the stained biofims was measured using a spectrophotometer.

### 2.8 Morphology of strains by transmission electron microscopy

*K. pneumoniae* was streaked on YPD agar; a single colony was activated in YPD medium for 8 h (37°C, 180 r/min). It was then transferred 1:100 to fresh YPD, shaken to logarithmic phase. One milliliter suspension (approximately 3.0 × 10^7^ CFU) was centrifuged, washed 3 times with PBS, fixed in 3% glutaraldehyde at 4°C overnight. 20 μL was loaded on a copper grid, stained with phosphotungstic acid for 1 min, air-dried, and observed via transmission electron microscopy.

### 2.9 Analysis of pathogenicity of strains

To establish a murine acute liver abscess model, male C57BL/6J mice (6–7 weeks old, Charles River Corp., Beijing, China) were randomly divided into 13 groups (*n* = 10 mice per group). Each group was inoculated intragastrically with *K. pneumoniae* isolates (10^7^ CFU/200 μL), with the reference strain NTUH-K2044 (at the same dose) and YPD broth (200 μL per mouse) as controls. The survival status of mice in each group was monitored and recorded continuously within 72 h. At the experimental endpoint, mice were anesthetized, and blood samples were collected for routine analysis. Subsequently, liver and lung tissues were harvested for hematoxylin-eosin (*H*&*E*) staining and histopathological examination.

### 2.10 Data availability

Whole-genome sequencing files were submitted to the National Center for Biotechnology Information^3^ (for specific genome accession numbers, please see Supplementary Table 1).

### 2.11 Statistical analysis

The survival curves of mice were plotted with GraphPad Prism (version 10.0) and statistically analyzed using the Log-rank test. For assessing differences between the two groups, a two-tailed *t*-test was performed. All data are shown as mean ± SD. Statistical significance was defined as *P* < 0.05.

## 3 Results

### 3.1 Characterization of genotypic variability and virulence gene patterns

This study characterized 11 clinical *K. pneumoniae* isolates (Table 1), revealing substantial genotypic diversity through serotyping and sequence typing (ST) analysis. The isolates were classified into six distinct serotypes, with the following distributions: K1 (3 isolates, including ST23, ST700, and one novel ST), K2 (2 isolates, ST65 and ST86), K5 (2 isolates, both ST60), K20 (1 isolate, ST420), K57 (2 isolates, both ST218), and K80 (1 isolate, harboring an additional novel ST). These strains exhibited notable variability in both serotypes and genomic backgrounds.

**TABLE 1:** General information of *Klebsiella pneumoniae* clinical isolates.

Isolate number	Serotype type	Sequence type	String test	Virulence genes									
				*magA*	*Aero-bactin*	*allS*	*kfu*	*ybtA*	*IroN*	*terW*	*iutA*	*rmpA*	*silS*
*Kpn*-S1-001	K1	ST23	+	+	+	+	+	+	+	+	+	+	+
*Kpn*-S1-003	K1	ST700	+	+	+	−	+	+	+	−	+	−	+
*Kpn*-S1-009	K1	New	+	+	+	+	+	+	+	+	+	+	+
*Kpn*-S2-029	K2	ST65	+	−	+	−	−	−	+	+	+	+	+
*Kpn*-S2-048	K2	ST86	−	−	−	−	−	−	−	−	−	−	−
*Kpn*-S5-036	K5	ST60	+	−	+	−	+	+	+	−	−	−	−
*Kpn*-S5-105	K5	ST60	−	−	+	−	+	+	+	−	−	−	−
*Kpn*-S20-067	K20	ST420	+	−	+	−	+	+	+	+	+	+	+
*Kpn*-S57-066	K57	ST218	+	−	+	−	−	+	+	+	+	+	+
*Kpn*-S57-077	K57	ST218	−	−	+	−	−	−	+	+	+	+	+
*Kpn*-S80-110	K80	New	−	−	−	−	−	−	−	−	−	−	−

In virulence gene analysis, the strains showed distinct lineage—specific distribution patterns: K1-ST23 (S1-001) and K1-novel ST (S1-009) carried all 10 target virulence genes and displayed positive string test results; K1-ST700 (S1-003) and K20-ST420 (S20-067) lacked *allS, terW, rmpA* and *magA, allS*, respectively, but retained most other virulence genes. K2-ST65 (S2-029) and K57-ST218 (S57-066/077) had unique deletion profiles. The K2-ST65 isolate exhibited deletions of *magA*, *allS*, *ybtA* and *kfu*, while the two K57-ST218 isolates maintained six intact virulence factors (*aerobactin*, *iroN*, *terW*, *iutA*, *rmpA*, and *silS*). Notably, *ybtA* was uniquely present in strain S57-066 but absent in S57-077. Consistent with the K2-ST65 strain, both K57-ST218 isolates lacked *magA*, *allS*, and *kfu* virulence determinants. K5-ST60 strains (S5-036/105) maintained only four core virulence genes (*aerobactin*, *kfu*, *iroN*, and *ybtA*), whereas K2-ST86 (S2-048) and the novel K80 (S80-110) strains showed complete absence of detectable virulence determinants.

Comparative analysis of virulence gene distribution among strains sharing the same serotype but differing in sequence types (STs) revealed distinct patterns: Within the K1 serotype, strains ST23 (S1-001) and novel ST (S1-009) exhibited complete sets of all ten tested virulence genes, whereas strain ST700 (S1-003) lacked *allS*, *terW*, and *rmpA*. Among K2 serotype strains, ST65 (S2-029) tested positive for *aerobactin*, *iroN*, *terW*, *iutA*, *rmpA*, and *silS* but negative for *magA*, *allS*, *kfu*, and *ybtA*. These findings demonstrate significant intra-serotype heterogeneity in virulence gene profiles across different STs, highlighting the importance of ST-level characterization in virulence assessment.

### 3.2 Biological characteristics of clinical *K. pneumoniae* isolates

**FIGURE 1:**
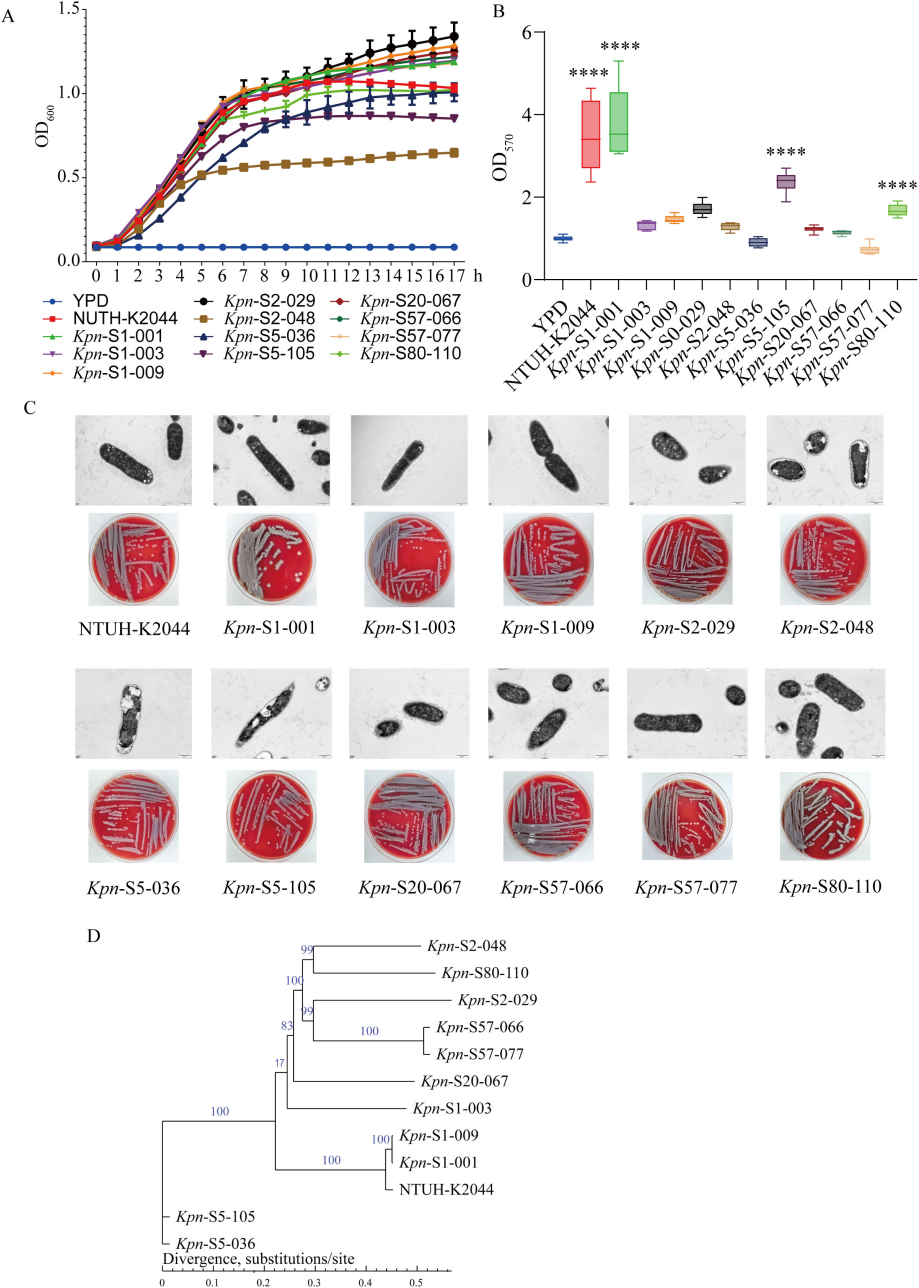
Characteristics of *K. pneumoniae* isolates. **(A)** Growth curves, *n* = 3. **(B)** Biofilm formation ability in 24 h, *n* = 6. ****P < 0.001. **(C)** The morphological characteristics of transmission electron microscopy and blood plate culture. **(D)** Phylogenetic tree of 11 isolates based on core SNPs, strain NTUH-K2044 was used as a reference.

As shown in Table 1, the string test yielded a distinct distribution across the tested strains, with seven isolates (S1-001/003/009, S2-029, S5-036, S20-067, and S57-066) demonstrating characteristic positive hypermucoviscosity (+), while four strains (S2-048, S5-105, S57-077, and S80-110) were consistently negative (−). Kinetic analysis of 11 clinical *K. pneumoniae* isolates revealed significant differences in growth performance (Figure 1A). Strain K2-ST65 (S2-029) exhibited the strongest proliferative capacity and an extended logarithmic phase. K1-novel ST (S1-009) and K57-ST218 (S57-077) showed similar growth rates, but the latter displayed a pronounced lag phase before 12 h. Strains with moderate growth capacity included K1-ST23 (S1-001), K1-ST700 (S1-003), K20-ST420 (S20-067), K57-ST218 (S57-066), and K80-novel ST (S80-110); they had a relatively stable peak OD_600_ values, though the timing of entering the stationary phase varied (earlier for S1-003, later for S80-110). Among strains with limited growth, K2-ST86 (S2-048) exhibited the weakest proliferative ability, while K5-ST60 (S5-105) and K5-ST60 (S5-036) displayed phenotypic heterogeneity due to early stagnation and prolonged lag phase, respectively. Notably, though S57-066 and S57-077 belonged to the same lineage (K57-ST218), the former grew faster. In summary, the growth kinetics of the 11 clinical *K. pneumoniae* isolates showed no direct correlation with virulence gene profiles (e.g., strongly proliferative S2-029 lacked *magA*/*allS*, while highly virulent gene-loaded strain S1-001 only exhibited moderate growth).

In biofilm formation assessment (Figure 1B), K1-ST23 (S1-001) demonstrated the strongest biofilm—forming capability, while K57-ST218 (S57-077) displayed the weakest. K5-ST60 (S5-105) and K80-novel ST (S80-110) exhibited relatively strong biofilm formation, other strains showed discontinuous differences—K1-ST700 (S1-003), K1-novel ST (S1-009), K2-ST65 (S2-029), K2-ST86 (S2-048), K20-ST420 (S20-067), whereas K5-ST60 (S5-036), and K57-ST218 (S57-066/077) were much weaker. These observations suggest that *K. pneumoniae* isolates sharing the same serotype but exhibiting different sequence types (STs) may display divergent biofilm—forming capacities, with K1 serotype isolates demonstrating notably stronger biofilm production. These findings highlight substantial diversity in virulence gene profiles, serotypes, and biofilm formation among *K. pneumoniae* strains, offering key insights into their distinct pathogenicity mechanisms.

Ultrastructural and colony morphology characteristic analyses (Figure 1C) revealed that all 11 *K. pneumoniae* isolates maintained a typical rod-shaped morphology and formed grayish-white non-hemolytic colonies, while exhibiting distinct polymorphism at the subcellular level. Eight clinical isolates (S1-001/003/009, S2-029, S20-067, S57-066/077, and S80-110) along with the reference strain NTUH-K2044 consistently exhibited uniform electron-dense bacilli with well-defined capsular structures under electron microscopy. In contrast, three strains (S5-036/105 and S2-048) displayed distinctive intracellular vacuole-like formations. Notably, despite ultrastructural heterogeneity, all strains consistently exhibited a conserved non-hemolysis phenotype on blood agar plates.

The 11 *K. pneumoniae* strains were subjected to whole-genome sequencing, core single-nucleotide polymorphisms (SNPs) were used to construct the phylogenetic tree (Figure 1D). The clustering analysis results were as follows: S5-105 and S5-036 formed an independent clade, distinctly separated from the other strains. The remaining strains are divided into two main subclades: Subclade 1 contained 7 strains including S1-003, S2-048/029, S80-110, S57-066/077, and S20-067; Subclade 2 includes S1-009/001, and NTUH-K2044. The degree of differentiation revealed that S5-105 and S5-036 exhibited a higher level of differentiation compared to the other strains.

Vitek 2 automated analysis system was used to evaluate the MICs of common antibiotics of 11 clinical *K. pneumoniae* strains. As shown in Table 2, overall resistance phenotype showed that 9 of 11 strains were pan-susceptible, with an overall low drug resistance rate. All strains were 100% sensitive to carbapenems (imipenem, meropenem), cephalosporins (ceftazidime, cefepime), aminoglycosides (amikacin, tobramycin), and monobactam (aztreonam). Among quinolones, S5-105 exhibited resistance to ciprofloxacin (MIC = 1 μg/mL) and intermediate susceptibility to levofloxacin (MIC = 1 μg/mL). Two isolates showed non-susceptible phenotypes to penicillins and β-lactamase inhibitors: S5-036 displayed intermediate susceptibility to sulbactam (MIC = 16 μg/mL), while S57-066 exhibited intermediate susceptibility to piperacillin (MIC = 32 μg/mL) and resistance to sulbactam (MIC ≥ 32 μg/mL). All other tested antimicrobial agents remained fully susceptible. This suggests that the studied *K. pneumoniae* population exhibits favorable susceptibility to most antimicrobial agents, with low resistance pressure.

**TABLE 2:** Antimicrobial susceptibility results of 11 *K. pneumoniae* strains.

Isolate number	MIC(μg/mL)											
	PIP	SUL	TAZ	AZM	CAZ	FEP	IPM	MEM	CIP	LEV	AMK	TOB
*Kpn*-S1-001	8	8	≤ 4	≤1	≤ 1	≤1	≤ 1	≤0.25	≤ 0.25	≤0.25	≤ 2	≤1
*Kpn*-S1-003	≤ 4	8	≤ 4	≤ 1	≤ 1	≤ 1	≤ 1	≤ 0.25	≤ 0.25	1	≤ 2	≤ 1
*Kpn*-S1-009	8	8	≤ 4	≤ 1	≤ 1	≤ 1	≤ 1	≤ 0.25	≤ 0.25	≤ 0.25	≤ 2	≤ 1
*Kpn*-S2-029	≤ 4	8	≤ 4	≤ 1	≤ 1	≤ 1	≤ 1	≤ 0.25	≤ 0.25	≤ 0.25	≤ 2	≤ 1
*Kpn*-S2-048	8	4	≤ 4	≤ 1	≤ 1	≤ 1	≤ 1	≤ 0.25	≤ 0.25	≤ 0.25	≤ 2	≤ 1
*Kpn*-S5-036	8	16	8	≤ 1	≤ 1	≤ 1	≤ 1	≤ 0.25	≤ 0.25	≤ 0.25	≤ 2	≤ 1
*Kpn*-S5-105	8	≤ 8	8	≤ 1	≤ 0.25	≤ 0.12	0.5	≤ 0.25	1	1	≤ 2	≤ 1
*Kpn*-S20-067	≤ 4	8	≤ 4	≤ 1	≤ 1	≤ 1	≤ 1	≤ 0.25	≤ 0.25	≤ 0.25	≤ 2	≤ 1
*Kpn*-S57-066	32	≥ 32	≤ 4	≤ 1	≤ 1	≤ 1	≤ 1	≤ 0.25	≤ 0.25	≤ 0.12	≤ 2	≤ 1
*Kpn*-S57-077	≤ 8	≤ 8	≤ 4	≤ 1	≤ 0.12	≤ 0.12	≤ 0.25	≤ 0.25	≤ 0.25	≤ 0.12	≤ 2	≤ 1
*Kpn*-S80-110	≤ 8	≤ 8	≤ 4	≤ 1	≤ 0.12	≤ 0.12	≤ 0.25	≤ 0.25	≤ 0.25	≤ 0.12	≤ 2	≤ 1
NTUH-K2044	≤ 8	≤ 8	8	≤ 1	≤ 0.12	≤ 0.12	≤ 0.25	≤ 0.25	≤ 0.25	≤ 0.12	≤ 2	≤ 1

### 3.3 Pathogenicity analysis of 11 clinical *K. pneumoniae* isolates using a mouse model

**FIGURE 2:**
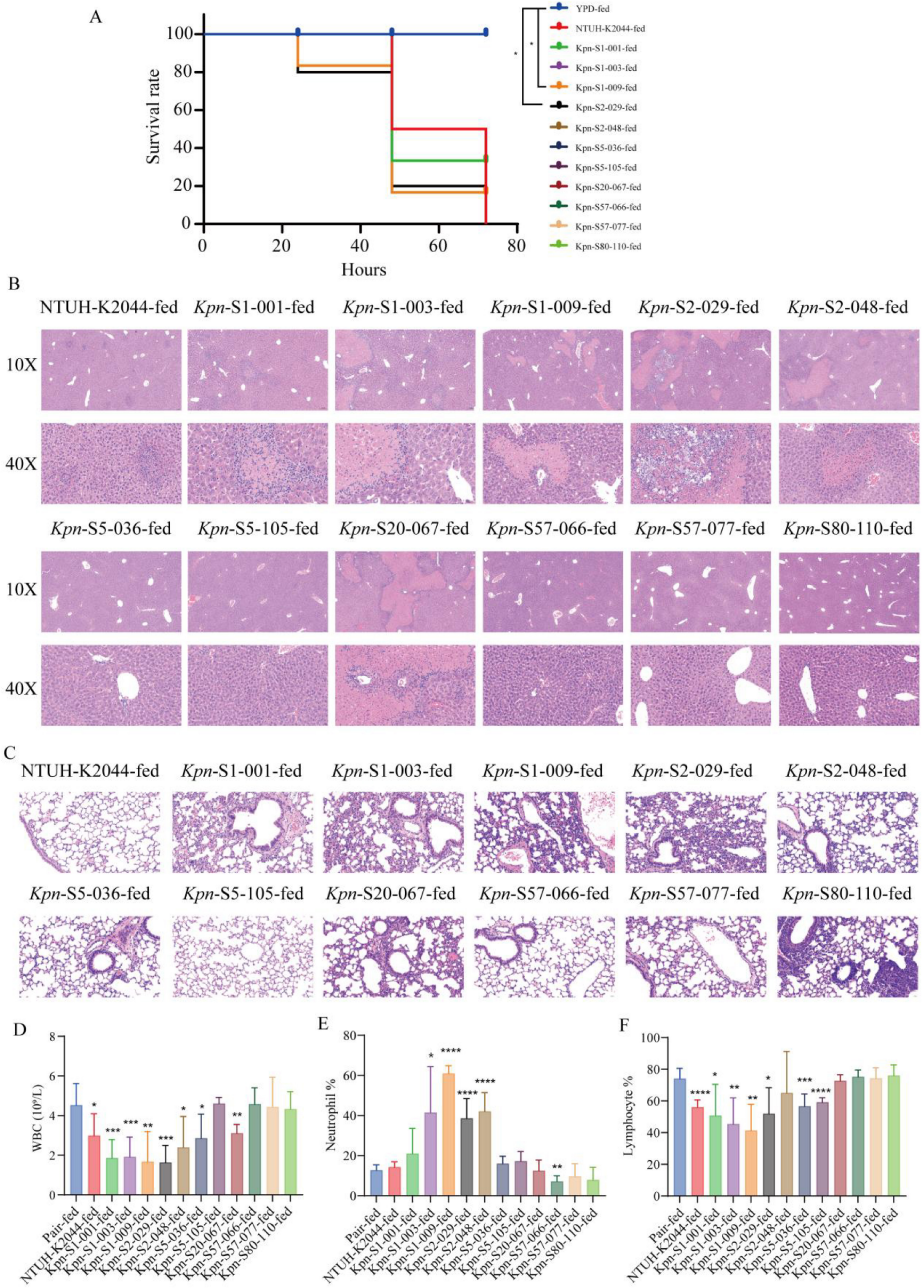
Pathogenicity analysis of 11 clinical *K. pneumoniae* isolates *in vivo*. **(A)** Survival rate analysis at 72 h post—infection in mice, *n* = 10. **(B)** *H&E* staining of liver tissue from infected mice. Microscopic examinations were carried out at magnifications of 10X and 40X, respectively. **(C)** Pathological staining of lung tissue from infected mice under a 40X magnification microscope. **(D–F)** Blood routine examination results, white blood cell count **(D)**, neutrophilic granulocyte percentage **(E)**, ratio of lymphocytes **(F)**. *P < 0.05, **P < 0.01, ***P < 0.005, ****P < 0.001.

We systematically evaluated the pathogenicity of 11 *K. pneumoniae* isolates in mice, revealing significant heterogeneity in virulence, manifested as a complex correlation between pathological damage severity and survival rates. The standard virulent strain NTUH-K2044 (72-h survival rate: 80%, Figure 2A) and highly pathogenic strains (e.g., S1-001/003, S2-029/048) induced typical suppurative liver damage, characterized by multifocal scattered abscess formation in hepatic lobules, accompanied by neutrophil infiltration, deposition of eosinophilic necrotic debris, and hepatocyte degeneration (ranging from swelling to coagulative necrosis) (Figure 2B). Among these, S1-009 and S2-029 exhibited the strongest pathogenicity (survival rate: 50%). In contrast, low—pathogenicity strains (S5-036/105, S57-066/077) caused only mild pathological damage; the novel strain S80-110 performed more different, with a 100% survival rate and no significant lesions. Notably, the pathology-survival correlation displayed specificity: while most strains showed a negative correlation between damage severity and survival rate (e.g., S1/S2 series), S20-067 triggered severe abscesses yet maintained a 100% survival rate, and S5-036/105 caused mild pathological damage but had only an 80% survival rate.

Next, we evaluated lung injury in infected mice, and the results revealed significant differences in pulmonary pathological damage caused by different *K. pneumoniae* isolates (Figure 2C). The highly pathogenic NTUH-K2044 strain exhibited near-normal alveolar architecture, with no detectable inflammatory infiltration. In contrast, most clinical strains induced varying degrees of lung injury: S1-001 and S80-110 led to severe alveolar destruction accompanied by extensive inflammatory consolidation; S2-029 and S2-048 displayed diffuse inflammatory infiltration and disruption of alveolar structure; S1-003 and S20-067 presented with alveolar septal thickened or focal tissue damage; and S5-036 showed only mild alveolar injury. Notably, S5-105 and S57-066 maintained intact alveolar structures with minimal inflammatory responses, which closely resembled the pathological features of uninfected control lung tissue. Of particular interest, strain K80-novel ST (S80-110), triggered severe pulmonary inflammatory responses. These findings highlight the complexity of tissue tropism and pathogenic mechanisms among different *K. pneumoniae* strains.

Blood test results revealed characteristic trends in mice infected with different *K. pneumoniae* isolates: highly pathogenic strains (NTUH-K2044, S1-001/003/009, S2-029) all induced significant decreases in white blood cells (WBC) (Figure 2D) and percentage of lymphocytes (LYM%) (Figure 2F), accompanied by increased percentage of neutrophils (NEU) (Figure 2E). Among them, S2-029 and K1-novel ST (S1-009) were particularly notable. This hematological pattern (WBC↓, LYM%↓, NEU%↑) was associated with severe inflammatory liver damage and significantly reduced survival rates. In contrast, strains including S2-048, S5-036/105, and S20-067 primarily caused selective decreases in WBC or LYM%, with no concurrent increase in NEU%. Some strains (S57-066/077, S80-110) showed essentially normal blood indicators. Notably, although the changes in blood tests generally correlated with pathogenicity, certain strains S20-067 exhibited inconsistencies between the severity of blood test abnormalities and liver damage, suggesting the potential existence of independent pathogenic mechanisms.

## 4 Discussion

This study provides a comprehensive analysis of 11 clinically isolated *K. pneumoniae* strains, revealing the complexity and diversity of pathogenic mechanisms within the *K. pneumoniae* population. Significant strain heterogeneity was observed across multiple dimensions: First, K1-ST23 (S1-001) and emerging K1 lineages (S1-009), as classical representatives of *K. pneumoniae*, demonstrated hypermucoviscosity and strong pathogenicity closely associated with intact virulence gene clusters (including *magA*, the aerobactin system, and *rmpA*/*rmpA2*, among others). Furthermore, the study highlights that K1-type strains exhibit higher conservation in their virulence plasmids and chromosomes compared to other types, which may facilitate their adaptation and spread across diverse environments (Dai et al., 2025). These genes synergistically (Hu et al., 2023) confer complete capsule biosynthesis capacity (Hunt et al., 2011; Yeh et al., 2010; Figure 1C), high-efficiency iron acquisition systems (Russo et al., 2011; Zhu et al., 2021), and potent immune evasion mechanisms (Sohrabi et al., 2022; Tu et al., 2024)—virulence determinants that collectively underlie severe invasive infections as demonstrated in multinational epidemiologic investigations.

Notably, the study observed that K2-ST65 strains (S2-029) exhibited pathogenic characteristics contradicting traditional assumptions: despite lacking canonical virulence genes like *magA* and *rmpA*, they retained high pathogenicity, challenging the “core virulence determinant” hypothesis. Their heightened virulence did not fully depend on known virulence genes or plasmids (e.g., the KP-06 strain carrying a pLVPK-like plasmid showed inconsistent virulence) (Fu et al., 2019), suggesting the existence of unidentified alternative pathogenic pathways (Sohrabi et al., 2022), possibly mediated by genome-specific adaptive features or compensatory mechanisms. This phenomenon is not isolated, as similar observations have been made in strains like CA-MRSA ST93-IV, which exhibit exceptional virulence despite limited virulence gene repertoires—potentially due to subtle regulatory gene modifications (Chua et al., 2011).

In-depth studies show that *K. pneumoniae* virulence regulation exhibits high environmental adaptability and plasticity, with phenotypic heterogeneity across three dimensions: (1) strain-level divergence under different conditions (Oh et al., 2025) (e.g., insertion sequence transposition), (2) genomic variations and environmental cues (temperature, pH, etc.) driving virulence gene heterogeneity (Barkovskii and Brown, 2024; Shi et al., 2025), and (3) uncharacterized metabolism-virulence coupling mechanisms modulating pathogenicity. For instance, *Listeria monocytogenes* employs *PrfA* to sense nutrient status and modulate virulence (Friedman et al., 2017), implying that bacteria may compensate for fitness deficits caused by the absence of classical virulence genes via metabolic rewiring or alternative pathways. Together, these findings converge on a central insight: *K. pneumoniae* pathogenicity is not solely determined by the presence or absence of specific virulence genes but emerges as a dynamic outcome shaped by multilayered regulatory factors, including genomic background, environmental stress, and metabolic remodeling.

Biofilm formation, an essential bacterial survival strategy, is governed by fimbrial genes (e.g., *csu* cluster in *A. baumannii*) and regulatory systems (*csgD* in *E. coli*, *mrkH* in *K. pneumoniae*) (Kishii et al., 2020). While canonical mechanisms involve components like *BfmR/S* systems in *A. baumannii* (Lee et al., 2020) or curli fibers in *E. coli* (Azam and Khan, 2022; Azam et al., 2020), our study identified the K80- novel ST strain S80-110 exhibiting relatively strong biofilm capacity despite lacking traditional virulence genes (*magA*, *aerobactin* etc.) (Table 1 and Figure 1B). This parallels emerging evidence of non-classical biofilm pathways, including plasmid—mediated formation in *E. coli* (Fang et al., 2021; He et al., 2021) and conditional biofilm production in KPC-2-producing *K. pneumoniae* (Naparstek et al., 2014), demonstrating the evolutionary plasticity of biofilm development independent of traditional virulence determinants.

Our study revealed that eight clinical isolates (S1-001/003/009, S2-029, S20-067, S57-066/077, and S80-110) and the reference strain NTUH-K2044 exhibited typical electron—dense bacillary morphology with well-defined capsular structures under transmission electron microscopy, while three strains (S5-036/105 and S2-048) displayed distinct intracellular vacuole-like structures (Figure 1C). The formation of these vacuolar structures likely involves two key mechanisms: First, aberrant release of outer membrane vesicles (OMVs) (Li et al., 2025), which contain various virulence—associated factors and antibiotic resistance determinants, plays multifaceted roles in pathogenicity, biofilm formation (as demonstrated in *Aeromonas* studies) (Seike et al., 2021), and host-pathogen interactions. Second, alterations in membrane homeostasis, particularly membrane permeability changes induced by destabilizing agents such as NMP (1-(1-naphthylmethyl)-piperazine) (Anes et al., 2019), may remodel bacterial microenvironment and consequently modulate growth regulatory networks.

Based on core SNP-based whole-genome sequencing and antibiotic susceptibility analysis, this study systematically elucidated the genetic evolutionary relationships and drug resistance characteristics of 11 *K. pneumoniae* strains. The phylogenetic tree (Figure 1D) revealed that the strains were clustered into three clades: S5-105 and S5-036 formed a distinct clade with high genetic differentiation, while the remaining strains were grouped into Subclade 1 (7 strains) and Subclade 2 (3 strains including NTUH-K2044). Drug resistance phenotypes (Table 2) showed an overall high susceptibility of this strain population: 9 strains were pan-susceptible, with 100% susceptibility to carbapenems, cephalosporins and other tested antibiotics; only the strains in the distinct clade (S5-105, S5-036) and S57-066 in Subclade 1 exhibited specific resistant/intermediate phenotypes (to quinolones, sulbactam and the like). This phenomenon strongly suggests a potential association between the degree of genetic differentiation and the acquisition of specific drug resistance phenotypes, as well as unique morphological traits (intracellular vacuolar structures under electron microscopy, Figure 1C). Strains in the distinct clade may have acquired resistance genes or developed relevant mutations under unique evolutionary pressures (e.g., antibiotic exposure), whereas the dominant subclades maintained high antibiotic susceptibility. This study not only clarified the genetic structure and low drug resistance background of the tested strains, but also uncovered the association between genetic differentiation and phenotypes, which provides important evidence for deciphering the evolutionary mechanisms of *K. pneumoniae*, as well as for the clinical precision prevention and control and rational use of antimicrobial agents against clinical *K. pneumoniae* infections.

Animal studies have demonstrated that the pathogenicity of *K. pneumoniae* exhibits significant strain specificity and tissue tropism. Strains like S1-001/003/009 and S2-029/048) can induce systemic liver injury and severe hematological abnormalities, while distinct strain characteristics (such as serotypes, sequence types, and virulence gene profiles) play decisive roles in determining the incidence and mortality of liver abscess. The K1/K2 capsule types dominate liver abscess pathogenesis through their antiphagocytic properties. Studies reveal that the K1 serotype inhibits C3b deposition (Al-Busaidi et al., 2024), thereby evading complement system attacks and increasing both the incidence and lethality of liver abscess. This characteristic has been further validated in animal models; for instance, in research on clinical *K. pneumoniae* isolates from Oman, K1-ST23 strains exhibited extreme pathogenicity in the Galleria mellonella model, with 50% of larvae dying within 24 h post-infection (Al-Busaidi et al., 2024).

The association between ST types and liver abscess is equally significant, with K1-ST23 emerging as one of the predominant pathogenic combinations globally due to its widespread prevalence. For example, a study in Barcelona, Spain, showed that K1-ST23 strains carrying *magA*/*rmpA* genes significantly increased the incidence of pneumonia and liver abscess in bacteremia patients (Cubero et al., 2016). In contrast, K2-type strains demonstrate marked heterogeneity in virulence, with some strains (e.g., K2-ST86) being less virulent while others (e.g., K2-ST65) exhibit strong invasive capabilities. Our data support this observation: the K2-ST65 strain (S2-029) displayed the fastest proliferation rate and induced the most severe liver injury in murine models, resulting in the lowest survival rate, whereas the K2-type K2-ST86 strain (S2-048) showed slower proliferation and a murine survival rate as high as 100%. This indicates that serotype-ST combinations not only influence bacterial immune evasion but also determine host immune defense efficiency, thereby affecting liver abscess incidence and clinical outcomes.

Although less studied, non-classical capsule types (e.g., K20, K57, K80) may still pose potential severe threats in liver abscess pathogenesis. The O-acetylation modification of the K80 capsule might interfere with host immune recognition, altering bacterial-immune system interactions and enhancing pathogenicity (Jiang et al., 2024). Our research identified that a novel ST80 strain (S80-110), while not causing significant liver tissue damage (Figure 2B), exhibited robust growth rates (Figure 1A), strong biofilm formation (Figure 1B), and notable pulmonary injury (Figure 2C)—a phenomenon consistent with existing reports, though its mechanisms require further exploration. Additionally, combinations of non-classical capsule types with specific STs (e.g., K57-ST218) may form highly lethal pairings, likely due to synergistic effects between genetic background and capsule properties, though current evidence remains limited.

Notably, capsule switching plays a pivotal role in the virulence evolution of *K. pneumoniae*. The *cps* gene cluster can undergo recombination via horizontal gene transfer, leading to capsule type variations that impact bacterial pathogenicity. For instance, studies on *Streptococcus pneumoniae* have confirmed that bacteria can acquire new capsular operons to generate strains with altered virulence (Sabharwal et al., 2014), and this mechanism may similarly operate in *K. pneumoniae*.

In summary, the pathogenicity of *K. pneumoniae* is highly complex, with strain-specific characteristics (e.g., capsule types, ST combinations, and virulence factors) collectively determining tissue—specific invasiveness, immune evasion efficiency, and ultimate clinical outcomes. Future research should further elucidate the pathogenic mechanisms of non-classical serotypes, the transmission risks of emerging STs, and the implications of capsule switching for antimicrobial therapy, thereby providing a more robust theoretical foundation for precision medicine in liver abscess management.

## 5 Limitations of study

This study has several limitations: (1). Functional validation of virulence genes requires further clarification through gene knockout experiments or transcriptomic analysis; (2). Molecular mechanisms of biofilm formation remain unresolved and warrant deeper investigation; (3). Drivers of tissue—specific pathogenicity need to be explored in greater depth. Future research should employ multi-omics integration analyses (e.g., genome-wide association studies or single-cell transcriptomics) to elucidate the molecular basis of hypervirulent *K. pneumoniae* phenotypic heterogeneity and provide insights for targeted prevention and control strategies.

## Data Availability

The original contributions presented in this study are included in this article/Supplementary material, further inquiries can be directed to the corresponding authors.

## Footnotes

1
https://bigsdb.pasteur.fr/klebsiella/

2
http://www.mgc.ac.cn/VFs/main.htm

3
https://submit.ncbi.nlm.nih.gov/
