# Supplementary material for: Pathogenic diversity of Klebsiella pneumoniae strains with different serotypes and sequence types from human liver abscess
Source: Front Microbiol. 2026 Mar 4;17:1730966. doi: 10.3389/fmicb.2026.1730966 (PMC12996106; doi:10.3389/fmicb.2026.1730966)
Supplement: Supplementary file 1 [file Table_1.docx]

**Supporting Information**

# Table S1. Accession number for whole-genome sequencing data.

| **Sample_name** | **Isolate_name** | **Accession number** |
| --- | --- | --- |
| *Kpn*-S1-001 | 10430 | JAJOPT000000000 |
| *Kpn*-S1-003 | 10668 | JAJOPV000000000 |
| *Kpn*-S1-009 | 11535 | JBUBKT000000000 |
| *Kpn*-S2-029 | 12956 | JAJOQV000000000 |
| *Kpn*-S2-048 | 14795 | JAJORP000000000 |
| *Kpn*-S5-105 | QD77 | JAJOXY000000000 |
| *Kpn*-S5-036 | 13329 | JAJORD000000000 |
| *Kpn*-S20-067 | 16101 | JAJOSI000000000 |
| *Kpn*-S57-066 | 15970 | JAJOSH000000000 |
| *Kpn*-S57-077 | QD77 | JAJOWC000000000 |
| *Kpn*-S80-110 | QD82 | JAJOYD000000000 |
